# Bonding Behavior and Quality of Pressureless Ag Sintering on (111)-Oriented Nanotwinned Cu Substrate in Ambient Air

**DOI:** 10.3390/ma17174423

**Published:** 2024-09-09

**Authors:** Xingming Huang, Wei He, Jialong Liang, Hao-Kun Yang, Chunliang Zhou, Zhi-Quan Liu

**Affiliations:** 1 Shenzhen Institute of Advanced Technology, Chinese Academy of Sciences, Shenzhen 518055, China; xm.huang@siat.ac.cn (X.H.); w.he@siat.ac.cn (W.H.); jl.liang@siat.ac.cn (J.L.); 2 Yantai Research Institute, Harbin Engineering University, Yantai 264000, China; chunliangzhou@hrbeu.edu.cn; 3 Shenzhen College of Advanced Technology, University of Chinese Academy of Sciences, Shenzhen 518055, China; 4 Smart Manufacturing Division, Hong Kong Productivity Council, Hong Kong SAR 999077, China; hkyang@hkpc.org

**Keywords:** pressureless Ag sintering, nanotwinned Cu, interfacial oxide, microstructure, bonding quality

## Abstract

(111)-oriented nanotwinned Cu ((111)nt-Cu) has shown its high surface diffusion rate and better oxidation resistance over common polycrystalline Cu (C-Cu). The application of (111)nt-Cu as an interface metallization layer in Ag-sintered technology under the role of oxygen was investigated in this work, and its connecting behavior was further clarified by comparing it with C-Cu. As the sintering temperature decreasing from 300 to 200 °C, the shear strength on the (111)nt-Cu substrate was still greater than 55 MPa after sintering for 10 min. The fracture surface correspondingly changed from the interface of Ag/die to mixed fracture mode, involving the interface of the Ag/Cu substrate and Ag/die. The existence of copper oxide provided a tight connection between Ag and the (111)nt-Cu substrate at all of the studied temperatures. Although lots of small dispersed voids were seen at the interface between copper oxide and (111)nt-Cu at 300 °C, these impurity-induced voids would not necessarily be a failure position and could be improved by adjusting the sintering temperature and time; for example, 200 °C/10 min or heating to 300 °C, and then start cooling at the same time. The microstructure of Ag-Cu joint on (111)nt-Cu behaved better than that on C-Cu. The thinner copper oxide layer and the higher connection ratio of the interface between copper oxide and Ag were still found on the (111)nt-Cu connection’s structure. The poor connection between copper oxide and Ag on C-Cu easily became the failure interface. By controlling the thickness of copper oxide and the content of impurity-induced voids, the use of (111)nt-Cu in advanced-packaging could be improved to a new level.

## 1. Introduction

With the emergence of wide band gap (WBG) semiconductors on power modules, high voltage and high temperature are common situations [1]. Huge heat flows introduce additional high-temperature reliability challenges, because the operation temperature of power modules can reach to above 250 °C [2]. As a newly developed bonding technology, sintered silver is becoming a promising interconnection solution for the die attachment of power modules. This technique possesses high electrical and thermal conductivity, low-temperature sinterability, and high operating temperature [3,4]. At present, bonding on bare Cu substrates, Ag-plating substrates and Au-plating substrates can acquire an acceptable bonding strength [5,6,7,8,9,10]. Despite the fact that better bonding quality can be realized on Ag- or Au-plating substrates, direct Cu bonding, which has many practical and financial incentives, still seems an ideal option. However, the easy oxidation of the Cu substrate during the sintering process is still a factor limiting the application of this substrate [11,12].

Up to now, several studies have demonstrated the enhancement of the Ag-Cu joint under air atmosphere by forming Cu_2_O [12,13,14,15,16]. Cu_2_O nanoparticles can be observed within the bonding joints, which was formed under the effect of solvent. Self-generated Ag nanoparticles were also found during the sintering. An intact Ag-Cu bonding interface was realized through these two nanoparticles because of their high surface energy [12]. In addition to the Ag-Cu metallic bond, the Ag-Cu_2_O chemical bond was regarded to be another connection mode of the Ag-Cu interface [14]. When sintering at 265 °C with the oxidation degree from 20 × 10^3^ to 200 × 10^3^ ppm, the strengthening effect of oxygen maintained a relative balance [15]. A complex structure containing Cu_n_O compositions might have formed during the oxidation process of 20 min [13]. The weakening phenomenon of Ag-Cu joint sintering at air atmosphere was ascribed to the occurrence of CuO and cracks or voids. Additionally, the removal of high-boiling-point organics in the Ag paste is necessary to enhance the densification of sintered Ag. The presence of oxygen can accelerate this process [15]. The establishment of a tight Ag-Cu interface involving copper oxide is still a promising solution for sintering on a copper substrate under air atmosphere.

As an under-bump metallization, (111)nt-Cu has been applied in the field of electronic packaging interconnections because of its higher tensile strength and electrical conductivity [17,18,19,20,21,22,23,24]. The calculated surface diffusivity of Cu atoms on the (111) plane is three to four orders of magnitude faster than other planes, i.e., (110) and (100) [25]. By using (111)nt-Cu, Cu-to-Cu direct bonding with a high bonding quality could be achieved at 200 °C for 30 min only under the pressure of 0.6 MPa [26]. Moreover, the phenomenon about the self-healing of Kirkendall voids on Sn/(111)nt-Cu interface during 170 °C aging was founded [23]. The shrinkage of voids promoting by (111) surface diffusion gave a new insight to adjust the interconnect interface for sintering. Compared to polycrystalline Cu, the lower surface energy of the (111) plane and smaller proportion of large-angle grain boundaries made the (111)nt-Cu exhibit the better oxidation resistance [27]. That phenomenon showed that (111)nt-Cu displayed a potential application prospect in sintering connection under air atmosphere. The relevant research on this point was not reported.

In this study, the bonding quality and microstructure of the Ag-Cu sintering joint on the (111)nt-Cu substrate under air atmosphere were studied. The effect of a sintering temperature from 200 to 300 °C on the Ag-Cu interface was investigated. The bonding mechanism was further clarified by comparing the experimental phenomena on a common C-Cu substrate.

## 2. Materials and Methods

### 2.1. Sample Preparation

Plating the (111)nt-Cu thin film (~25 μm) was fabricated on polycrystalline C-Cu through direct-current electrodeposition. The contamination on the C-Cu’s surface was removed using acid before plating. The description of detailed electroplating methods for (111)nt-Cu was shown in our previous work [23]. To keep the similar roughness and remove the surface stress layer before sintering, after grounding by abrasive papers of #5000, the plating (111)nt-Cu and polycrystalline C-Cu were treated using electrolytic polishing.

A mature commercial Ag paste was used in this work to reduce the additional effect introduced by the Ag paste, and it consisted of four kinds of particles or flakes, i.e., micron-sized Ag flakes (average diameter of 1.83 μm, 12%) and spherical particles (average diameter of 1.68 μm, 11.5%), sub-micron-sized spherical particles (average diameter of 0.27 μm, 13.3%), and nano-sized spherical particles (average diameter of 0.06 μm, 63.2%).

Stencil printing was adopted for the preparation of silver film, and common polycrystalline Cu dummy chips with the size of 3 × 3 mm^2^ were finally placed on the as-printed silver films to make sandwich-like structure samples. The common polycrystalline Cu dummy chip has the same structure and surface characteristics as the C-Cu substrate. The prefabricated samples were sintered in a sintering furnace at temperatures of 200, 250, and 300 °C for 10 min, or other significant times, under a pressure-less condition in an air atmosphere. Preheating at 150 °C for 10 min was necessary to reduce the effect of the solvent in Ag paste.

### 2.2. Characteristics Analysis

An X-ray diffractometer (XRD, D8 Advance, Bruker Corporation, Berlin, Germany) with Cu K_α_ radiation and a focused ion beam (FIB, Helios 5 UX, Thermo Fisher Scientific, Waltham, MA, USA) was used to investigate the surface grain structure and the cross-section morphology of Cu substrates. The microstructure and composition determination of the Ag-Cu joint, as well as the fracture surface, were conducted on a field-emission scanning electron microscope (FESEM, Apreo 2, FEI Company, Hillsboro, OR, USA). The shear strength of the joint was measured using a DAGE 4000 with a shear height and shear speed of 50 μm and 50 μm/s, respectively.

The samples required for microstructure characterization were firstly ground with abrasive papers (2000# to 5000#), then polished with Al_2_O_3_ polishing fluid, and finally treated by argon ion beam milling (Ilion II 697, Ametek Inc., Berwyn, PA, USA) to obtain the actual morphology of Ag-Cu sintering joints and remove the surface stress layer. ImageJ software (Image-Pro Plus 6.0) was utilized to quantized the interface connection ratio on Ag-Cu joints.

## 3. Experimental Results

### 3.1. Ag-Cu Joint at 300 °C

**Figure 1:**
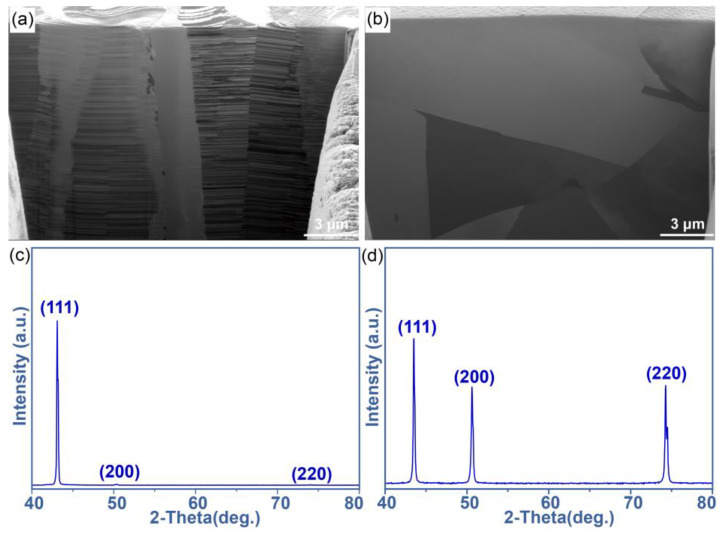
Cross-sectional FIB images and XRD patterns of (**a**,**c**) (111)nt-Cu and (**b**,**d**) C-Cu substrates.

The cross-sectional observation on both substrates showed the difference in their microstructure, as presented in Figure 1a,b. Columnar crystals with high-density parallel twins were seen on the (111)nt-Cu substrate, while irregular grain growth behavior was found on the C-Cu substrate. The grain sizes of both substrates were of the same order of magnitude. And the proportion of twin boundary of (111)nt-Cu substrate was extremely higher than that of the C-Cu substrate. The strong (111) preferred orientation on the (111)nt-Cu substrate is displayed in Figure 1c through XRD determination, which was calculated to be about 95 ± 3%. Cu grains with random orientations existed on the C-Cu substrate, as shown in Figure 1d.

**Figure 2:**
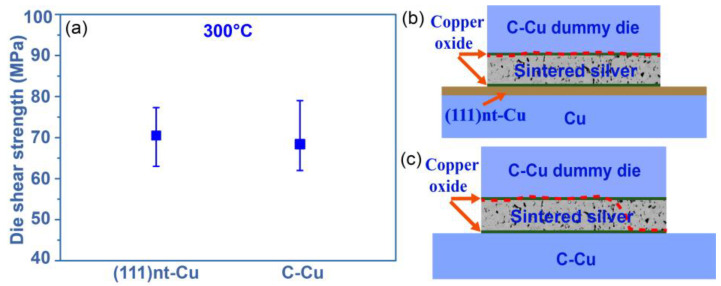
(**a**) Die shear strength and (**b**,**c**) fracture mode of (111)nt-Cu and C-Cu substrates sintered at 300 °C for 10 min.

**Figure 3:**
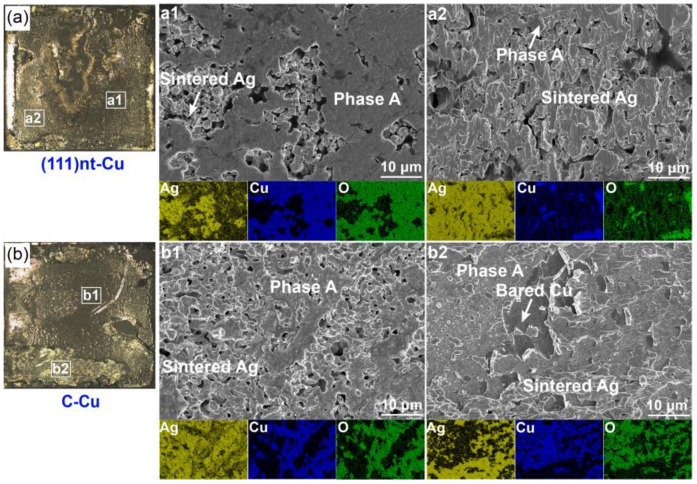
Die fracture surface with corresponding element mapping of (**a**) (111)nt-Cu and (**b**) C-Cu substrates sintered at 300 °C for 10 min. (**a1**,**a2**) and (**b1**,**b2**) are magnified views of local areas in (**a**) and (**b**), respectively.

To evaluate the sintering quality, the shear strength of the Ag-Cu joint after sintering at 300 °C on both substrates was determined. As illustrated in Figure 2a, the bonding strength of the (111)nt-Cu substrate was about 70.4 MPa, which was similar to that on the C-Cu substrate. By analyzing the fracture surface, it is found that the adhesion fracture mode happened on both substrates (the red dotted line in Figure 2b,c). The weaker point on the (111)nt-Cu substrate was the interface between the Cu dummy chip and the sintered Ag layer (Figure 2b), indicating the stronger adhesion quality of the interface between (111)nt-Cu and the sintered Ag layer. The magnified views in the local area of Figure 3a were shown in Figure 3a1,a2. Combined with the elements mapping, O and Cu coexisted in phase A, suggesting the formation of copper oxide. The main constituent phase in Figure 3a1 was copper oxide, while only a small amount of copper oxide could be observed in Figure 3a2. The failure occurred alternately at the interfaces of the Cu dummy chip/copper oxide and copper oxide/sintered Ag layer. The similar case was observed on the C-Cu substrate, as displayed in Figure 3b. It is found that the main position of fractures still happened at the interface of the Cu dummy chip/copper oxide or copper oxide/sintered Ag layer (Figure 3b1). The difference is that a fracture region passing through the interface between the C-Cu substrate and sintered Ag layer can be seen in Figure 3b2. The residual elongated sintered Ag, copper oxides, and bare Cu surface coexist in Figure 3b2.

**Figure 4:**
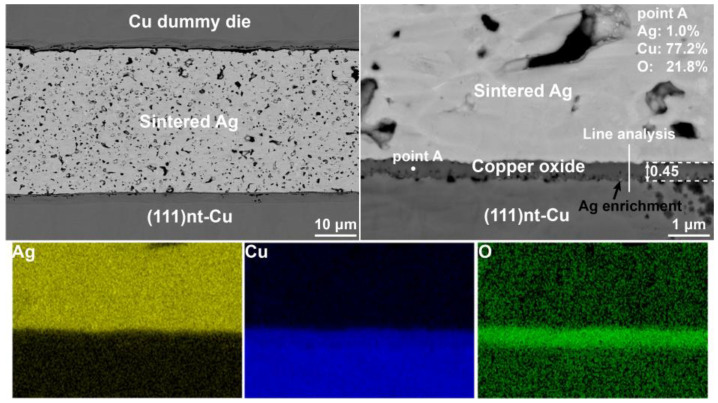
BSE images and EDS mapping of Ag-Cu joint on (111)nt-Cu substrate sintered at 300 °C for 10 min.

**Figure 5:**
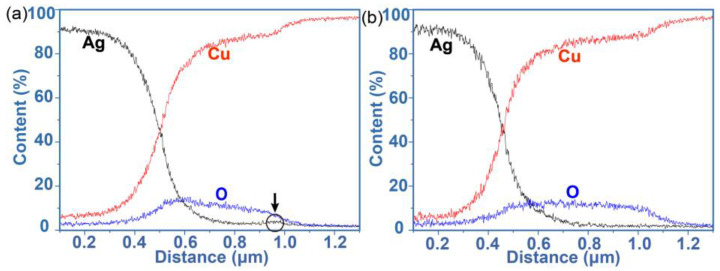
Compositional curves of Ag-Cu joint sintered at 300 °C for 10 min along the diffusion direction, (**a**) (111)nt-Cu and (**b**) C-Cu substrates. The enrichment phenomenon of Ag in (**a**) was highlighted with a circle indicating by black arrow.

**Figure 6:**
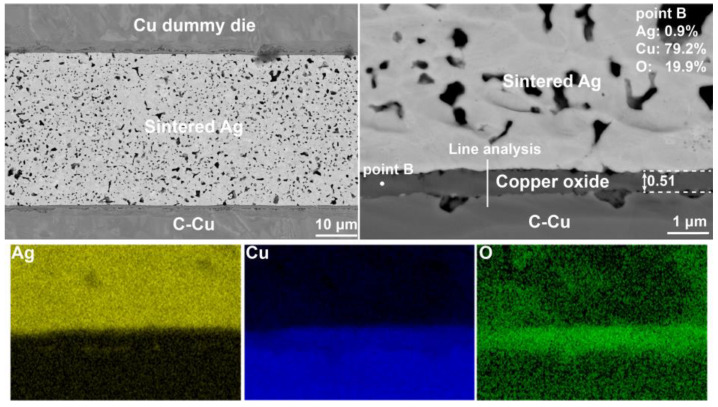
BSE images and EDS mapping of Ag-Cu joint on C-Cu substrate sintered at 300 °C for 10 min.

The backscattered electron (BSE) image at the cross-section of the Ag-Cu joint after sintering at 300 °C is presented in Figure 4. The sintered Ag layer with a uniform and microporous continuous bulk-like structure was formed during the sintering process. Most porous were in micrometer scale. As shown in Figure 4, a phase layer with mean thickness of 0.45 μm was seen at the interface between the sintered Ag and (111)nt-Cu substrate. Through an energy dispersive spectroscopy (EDS) analysis, Cu and O were the main constituent compositions in this phase. The content ratio between these two elements was about 4:1, indicating that this phase layer was the complex structure. The line analysis (Figure 5a) along the Ag-Cu interface confirmed that the composition of Cu and O in the copper oxide layer changed gradually. CuO and Cu_2_O nanocrystals coexisting within the copper oxide layer during the oxidation process were the common phenomenon [13,28]. Lots of small holes were seen within copper oxide layer adjecting to the (111)nt-Cu substrate, but that phenomenon was not found in the copper oxide layer closing to the Ag layer. A tight connection between the copper oxide layer and the Ag layer could be obtained. The formation of the tiny holes should be related to the impurity elements in plating (111)nt-Cu [29]. Additionally, enrichment of Ag at the edge of the oxide layer close to the Cu substrate occurred (Figure 4), as confirmed by Ag distribution in Figure 5a (the black arrow). Compared to the microstructure on C-Cu substrate in Figure 6, the thickness of the oxide layer on the (111)nt-Cu substrate was about 0.06 μm thinner (the O distribution in Figure 5a,b also confirmed this). The connecting quality between the copper oxide layer and the Ag layer on the C-Cu substrate was poorer than that on the (111)nt-Cu substrate, since the connecting ratio on the C-Cu substrate was about 60%, while that on the (111)nt-Cu substrate was about 70%. Despite the fact that small voids were not seen within the copper oxide layer on the C-Cu substrate, some huge holes with their sizes on the sub-micrometer level existed in the interface between the copper oxide layer and the C-Cu substrate. The occurrence of a bare Cu surface in Figure 3b2 suggests that these large voids might become failure points under mechanical stress. The microstructure of the Ag-Cu joint on the Cu dummy chip side was similar to that on the C-Cu substrate, and will not be described in detail hereafter. Therefore, although the connecting structure on both substrates showed a similar shear strength, by combining the fracture mode, the Ag-Cu joint on the (111)nt-Cu substrate at 300 °C should behaved better than that on the C-Cu substrate.

### 3.2. Ag-Cu Joint at 250 °C

**Figure 7:**
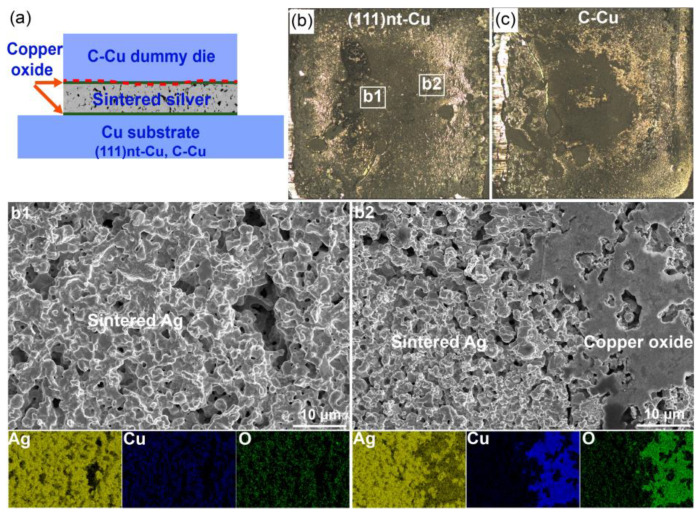
(**a**) Fracture mode and (**b**,**c**) fracture surface with corresponding element mapping of (111)nt-Cu and C-Cu substrates sintered at 250 °C for 10 min. (**b1**,**b2**) are magnified views of local areas in (**b**).

Figure 7 shows the fracture mode and fracture surface of (111)nt-Cu and C-Cu substrates after sintering at 250 °C. At this temperature, the adhesion fracture mode also happened (the red dotted line in Figure 7a). By analyzing the enlarged image of fracture surface in Figure 7b, the interface of the Cu dummy chip/copper oxide or copper oxide/sintered Ag layer was still the weaker point at 250 °C. Through the element mapping, the main phase in Figure 7b1 is sintered Ag, accompanied by a very small amount of copper oxide. The constituent phases in Figure 7b2 are copper oxide and sintered Ag. The interfaces of the Cu dummy chip/copper oxide and copper oxide/sintered Ag layer on the chip side were the weak positions. A similar phenomenon could be seen on the C-Cu substrate (Figure 7c), which resulted the same shear strength of connection structure on both substrates, i.e., about 56.4 MPa.

**Figure 8:**
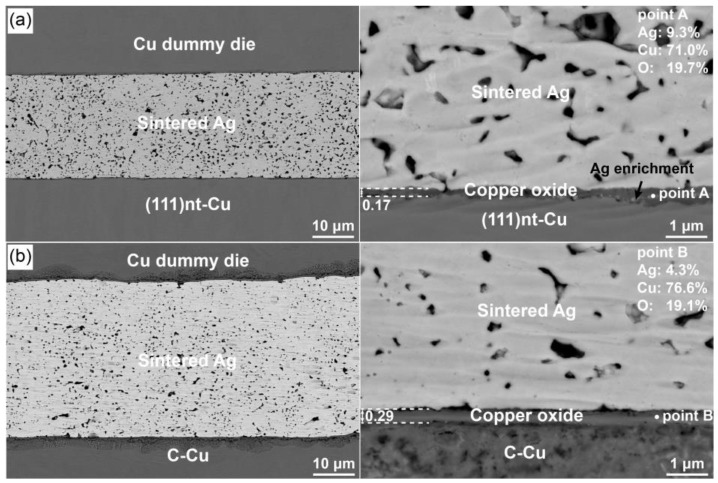
Morphology of Ag-Cu joint on (**a**) (111)nt-Cu and (**b**) C-Cu substrates sintered at 250 °C for 10 min.

The microstructure of the Ag-Cu joint after sintering at 250 °C was further investigated. As illustrated in Figure 8, the copper oxide layer could also be seen on the Ag-Cu interface, and the average thickness of this layer on the (111)nt-Cu and C-Cu substrates was 0.17 and 0.29 μm, respectively. The small void occurring at 300 °C on (111)nt-Cu was not apparent at this temperature. A tight connection between copper oxide and (111)nt-Cu with a small number of voids could be achieved; so did that for the interface between copper oxide and sintered Ag. The aggregation of Ag in the copper oxide layer was more obvious on the (111)nt-Cu substrate at 250 °C. The diffusion of Ag to the interface between copper oxide and Cu could improve the adhesion of Ag-Cu. The connecting quality on the C-Cu substrate at 250 °C was similar to that at 300 °C, apart from the disappearance of the large hole. Comparing the thickness of the copper oxide layer as well as the interfacial connection ratio of the Ag/copper oxide layer and the copper oxide layer/Cu substrate, the bonding microstructure on the (111)nt-Cu substrate at 250 °C should be better than that on the C-Cu substrate.

### 3.3. Ag-Cu Joint at 200 °C

**Figure 9:**
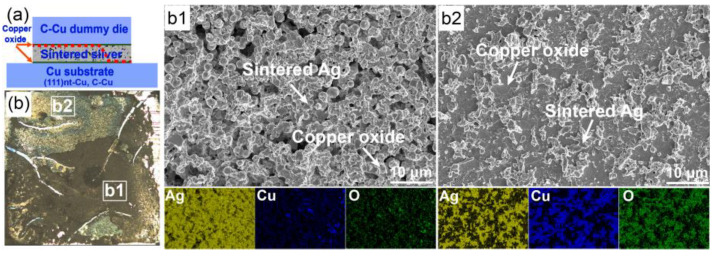
(**a**) Fracture mode and (**b**) fracture surface with corresponding element mapping of (111)nt-Cu substrate sintered at 200 °C for 10 min. (**b1**,**b2**) are magnified views of local areas in (**b**).

The sintering behavior of the Ag-Cu joint at 200 °C was also studied. The shear strength of the (111)nt-Cu substrate was determined to be about 61.0 MPa, presenting a mixed fracture mode (the red dotted line in Figure 9a). Two typical fracture locations in Figure 9b were further determined using EDS, as shown in Figure 9b1,b2. The fracture region b1 was the interface of sintered Ag/Cu dummy chip, accompanied by some residual discontinuous copper oxide phase. The other fracture region b2 was identified to be the interface of the sintered Ag/Cu substrate. In addition to the adhesive sintered Ag, copper oxide could also be seen under the sintered Ag layer. The same fracture mode could be found on the C-Cu substrate. Considering the influence of error, the shear strength on both substrates was close to the same level (i.e., 60~65 MPa).

**Figure 10:**
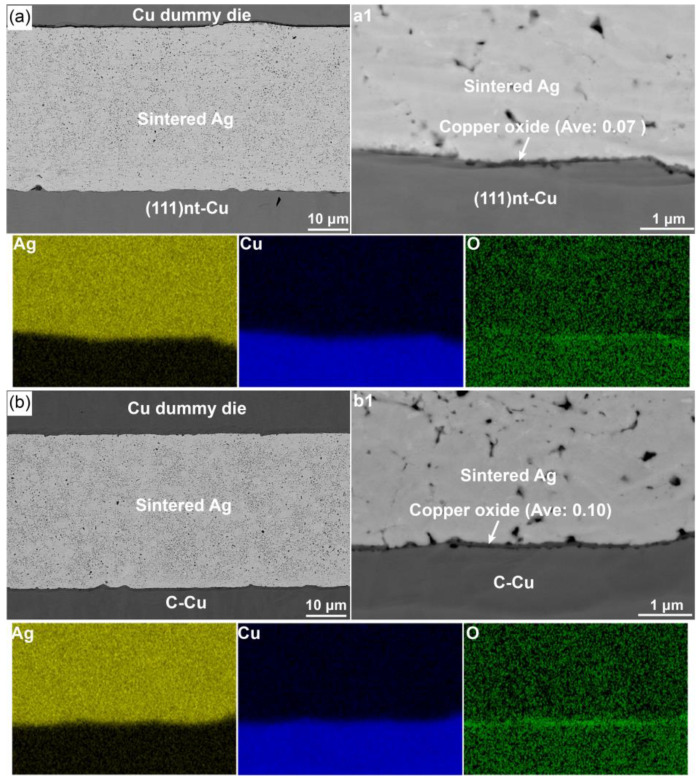
Morphology and EDS mapping of Ag-Cu joint on (**a**) (111)nt-Cu and (**b**) C-Cu substrates sintered at 200 °C for 10 min. Ave: Average thickness of copper oxide. (**a1**,**b1**) are magnified views of local areas in (**a**,**b**), respectively.

Figure 10 shows the microstructure of Ag-Cu joints on these two substrates. it is seen that the size of the hole in the Ag layer at this temperature was smaller and the Ag layer closing the Cu substrate displayed a higher densification. Under the air atmosphere, a dense Ag layer with fine void was acquired at 200 °C. The role of oxygen facilitating the sintering of Ag was more effective at this temperature. After sintering, a thin copper oxide with an average thickness of 0.07 μm was formed on the (111)nt-Cu substrate. The existence of the copper oxide contributed a tight connection between the sintered Ag and (111)nt-Cu. Being different to 250 and 300 °C, only a few small voids were found at the interface between copper oxide and the Cu substrate at 200 °C (Figure 10a1). Ag and Cu could still contact each other directly after sintering for 10 min. The connecting quality on the (111)nt-Cu substrate at 200 °C was improved obviously. By using ImageJ, the thickness of the copper oxide formed on the C-Cu substrate was calculated to be about 0.10 μm, higher than that on the (111)nt-Cu substrate. Some holes could be seen at the interface of Ag and copper oxide (Figure 10b1), but the number of these holes decreased correspondingly because of the enhancement of the Ag-sintered layer. Based on the microstructure of Ag-Cu interface at 200 °C, improvement of the sintering interface is possible by controlling the sintering temperature and time.

## 4. Discussion

**Figure 11:**
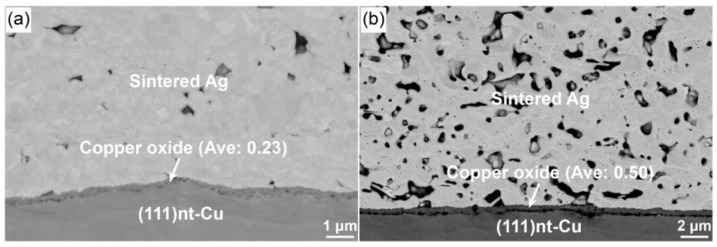
Morphology of Ag-Cu joint on (111)nt-Cu substrate sintered at 300 °C for (**a**) 0 and (**b**) 30 min. Ave: Average thickness of copper oxide.

The formation of the fine void at the interface between copper oxide and the nt-Cu substrate did not make this position to be the weakest point in the interconnect structure. That might be related to the phenomenon of these tiny voids dispersed in the copper oxide layer. During the plating process, the additive-dependent impurities, such as N, C, Cl, and S, often involved at the substrate-side or surface-side [29,30]. The impurity-induced voids in Figure 4 were caused by the out-diffusion and concentration of the above impurities. Thus, the sintering temperature and time had a significant effect on the diffusion of impurities. As the sintering temperature changing from 200 to 300 °C, the number of the fine voids within copper oxide increased obviously. So did that for the increasing of sintering time. When the samples were heated to 300 °C and then start-cooled at the same time, a tight connection between copper oxide and Cu substrate was acquired, as shown in Figure 11a. At this moment, only a few small voids were visible on the interface. As the sintering time increased to 10 min (Figure 4), the formation rate of voids increased with them dispersed on the interface. When the sintering time at 300 °C was prolonged to 30 min, these tiny voids gathered at the interface to form a delamination phenomenon, as presented in Figure 11b, and further became the fracture surface during the shear test. The appearance of delamination prevented the diffusion of Cu atoms, thus slowing the growth rate of copper oxide. However, by controlling the sintering temperature and time, a better Ag-Cu joint could be realized by using (111)nt-Cu under air atmosphere. Based on the current work, the results on the condition of “200 °C/10 min” or “heating to 300 °C and then start cooling at the same time” were recommended as the selection of sintering process. Additionally, another method to improve the bonding quality is to recommend controlling the type and content of impurities during plating. Exploration of the improvement of the process for the fabrication of (111)nt-Cu is necessary in future work.

**Figure 12:**
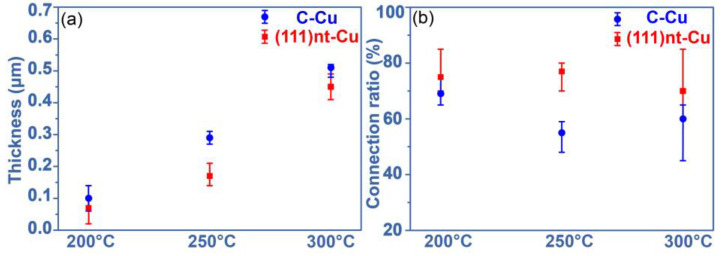
(**a**) Thickness of copper oxide and (**b**) connection ratio of the interface between Ag and copper oxide on (111)nt-Cu and C-Cu substrates.

By comparing the results obtained from (111)nt-Cu and C-Cu, two advantages were displayed in the former. One is that the oxidation rate on (111)nt-Cu during the sintering process was slower than that on C-Cu. Figure 12a shows the average thickness of copper oxide on the interface between the Ag and Cu substrate after sintering at different temperatures. The thickness of copper oxide on the (111)nt-Cu substrate was always thinner than that on the C-Cu substrate, indicating that the resistance to oxidation for (111)nt-Cu was excellent relative to C-Cu. (111)nt-Cu poses a high-content (111) orientation and high-density twin structure. The surface energy of the (111) plane was lower than that of other planes [31], which need higher activation energy for oxidation. Moreover, the existence of a twin boundary produced lots of triple points by intersecting with the grain boundaries [32]. These triple points would slow down the diffusion of Cu atoms. Additionally, the proportion of low-angle grain boundaries in our plating of (111)nt-Cu was lower than that in C-Cu, which might also limit the oxidation rate of Cu [27]. The other advantage is that the connecting ratio between copper oxide and the Ag layer was higher on the (111)nt-Cu substrate. The calculating connecting ratio after sintering at different temperatures is presented in Figure 12b. For the (111)nt-Cu substrate, the connecting quality between copper oxide and the Ag layer at 250 and 300 °C was significantly better than that of the C-Cu substrate. The Cu atom on the (111) plane has the fastest surface diffusion coefficient [25], resulting in a high diffusion rate of Cu atoms on the surface of (111)nt-Cu. That made more Cu atoms react with Ag atoms to form a thin interdiffusion region at the initial sintering stage. All an all, according to the copper oxide and its bonding quality with the Ag layer, the behavior of (111)nt-Cu in the sintering connection was superior to C-Cu.

At the studied temperatures, the shear quality on the (111)nt-Cu substrate was greater than 55 MPa. Along with the temperature decreasing, the fracture mode on the (111)nt-Cu substrate changed from the interface of the sintered Ag/Cu dummy chip to the mixed interfaces of the sintered Ag/Cu dummy chip and sintered Ag/Cu substrate. More specifically, the failure happened at the connecting interface related with copper oxide. The C-Cu was used as a dummy chip in this work. The oxidation behavior and microstructure of the Ag-Cu joint on the chip side were similar to that on the C-Cu substrate side. As described above, the microstructure of the Ag-Cu joint on the C-Cu substrate at 300 and 250 °C was apparently weaker than that on the (111)nt-Cu substrate, which resulted in the failure on the chip side of the sintered Ag/(111)nt-Cu interconnected structure. However, despite the thinner copper oxide layer and better connecting ratio of sintered Ag/copper oxide on the (111)nt-Cu substrate at 200 °C, the microstructure of the Ag-Cu joint on the C-Cu substrate at this temperature also behaved well. Thus, the occurrence of a mixed-fracture mode happened at 200 °C on the (111)nt-Cu substrate.

**Figure 13:**
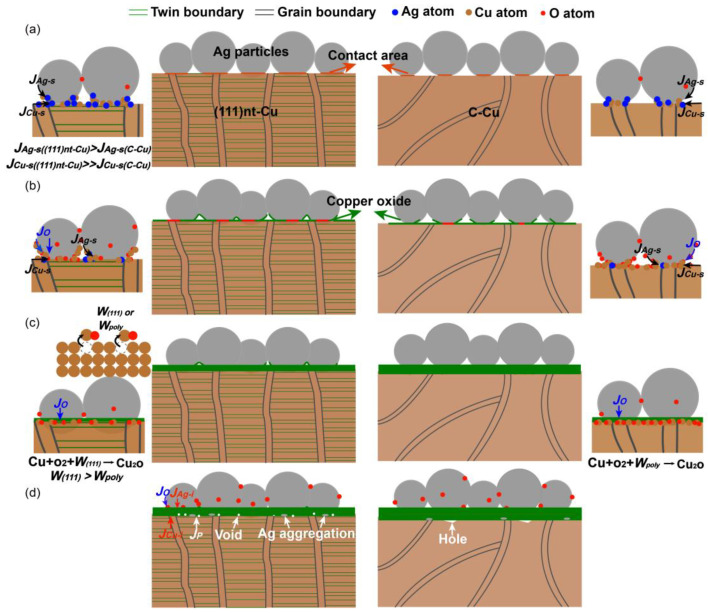
Schematic illustration of diffusion bonding of the Ag-Cu joint on (111)nt-Cu and C-Cu substrates, (**a**,**b**) the initial sintering stage, (**c**) the intermediate sintering stage, (**d**) the final sintering stage. *J*_*Cu-s*((111)*nt-Cu*)_: surface diffusion flux of Cu on (111)nt-Cu, *J*_*Cu-s*(*C-Cu*)_: surface diffusion flux of Cu on C-Cu, *J_Cu-i_*: interior diffusion flux of Cu, *J_p_* and *J_O_*: diffusion flux of impurity elements and O. *W*_(111)_ or *W_poly_*: the energy of Cu atoms escaping from (111) crystal lattice on (111)nt-Cu or other crystal lattices on C-Cu.

Figure 13 depicts the schematic of the sintering process of (111)nt-Cu under an air atmosphere. The surface and interior diffusion flux for Cu and Ag was, respectively, denoted as *J_Cu-s_*, *J_Ag-s_*, *J_Cu-i_*, and *J_Ag-i_*. *J_O_* represents the diffusion flux of O. At the initial sintering stage (Figure 13a), because of the higher *J_Cu-s_* on (111)nt-Cu, the Cu atoms supported by *J_Cu-s_* reacted with the Ag atoms to realize a tighter contact between the Ag and Cu. The role of surface diffusion for Ag was similar on both substrates to promote interconnection. But, owing to the existence of twinned boundaries on the surface of (111)nt-Cu, the *J_Ag-s_* on (111)nt-Cu should be slightly higher than that on C-Cu. As the temperature or time increased, the O atoms diffused along the Ag grains or the surface of holes (i.e., *J_O_* increased) and gathered on the surface of the Cu substrate to form copper oxide, as illustrated in Figure 13b. At this moment, the active Cu atoms supported by *J_Cu-s_* diffused along the surface of the Ag particles and then intersected with the O atoms to form copper oxide. However, that migration phenomenon was slow for C-Cu because of the lower *J_Cu-s_*, resulting in the relatively better connecting ratio between the sintered Ag and copper oxide on (111)nt-Cu. When a thin copper oxide film covered the surface of Cu (Figure 13c), the diffusion of Cu and Ag atoms was mainly controlled not by *J_Cu-s_* and *J_Ag-s_*, but by *J_Cu-i_* and *J_Ag-i_*. Due to the lower surface energy of the (111) plane, the escape of Cu atoms from the (111) crystal lattice to react with O were difficult and inhibited the growth of copper oxide. As the copper oxide had grown to a certain thickness, the tiny voids occurred because of the migration of purities (*J_p_*) from the Cu plating to the copper oxide layer, as seen in Figure 13d.

## 5. Conclusions

In this work, the change of microstructure of Ag-Cu sintering joints on (111)nt-Cu in the temperature range of 200–300 °C was studied. The connecting mechanism on this substrate was analyzed by comparing the bonding quality on C-Cu.

(1) The shear strength on (111)nt-Cu after sintering at 200 °C for 10 min was about 61.0 MPa, lower than that at 300 °C for 10 min. The bonding strength at all of the studied temperatures was within the acceptable range (≥55 MPa).
(2) The thickness of copper oxide on (111)nt-Cu was thinner than that on C-Cu, e.g., 0.17 μm on (111)nt-Cu vs. 0.29 μm on C-Cu at 250 °C, indicating the better oxidation resistance for (111)nt-Cu.
(3) The higher connection ratio of interface of Ag/copper oxide was determined on (111)nt-Cu after 10 min of sintering. Meanwhile, the interface of the copper oxide/Cu substrate showed a tight connection on both substrates, besides at 300 °C/10 min on C-Cu. The better connecting quality could be acquired on (111)nt-Cu after 10 min’ sintering.
(4) Longer sintering time or higher sintering temperature increased the amount of small voids in the interface of copper oxide/(111)nt-Cu. However, these dispersive small voids would not induce failure during the shear test unless the aggregation of them happened to produce delamination. Controlling the sintering time and temperature or controlling the number of impurities on (111)nt-Cu is an efficient way to further improve the occurrence of voids. Sintering at 200 °C for 10 min might be considered to be one of the optimal parameters.
(5) The fracture surface on (111)nt-Cu varied from Ag/die at 300 °C to mixed fracture mode involving the Ag/Cu substrate and Ag/die at 200 °C. The specific failure position happened at the interface between Ag/copper oxide or copper oxide/Cu on both connection structures. That was related with the poor connection between Ag/copper oxide or the formation of huge holes in the interface of copper oxide/C-Cu.

## Data Availability

The original contributions presented in the study are included in the article, further inquiries can be directed to the corresponding author.
